# Purtscher-like retinopathy following coronary artery bypass grafting in an antiphospholipid syndrome patient: a case report

**DOI:** 10.1186/s12886-023-02935-z

**Published:** 2023-05-04

**Authors:** Ahmed Ameen Ismail, Heba Eid Tolba, Sherin Hassan Sadek, Ragai Magdy Hatata

**Affiliations:** 1 Department of Ophthalmology, Faculty of Medicine, Fayoum University, Al Fayoum, Egypt; 2 Department of Rheumatology, Faculty of Medicine, Fayoum University, Al Fayoum, Egypt

**Keywords:** Purtscher retinopathy, Purtscher-like retinopathy, Coronary artery bypass grafting, Antiphospholipid syndrome, Case report

## Abstract

**Background:**

Purtscher retinopathy is a rare occlusive microangiopathy comprising a constellation of retinal signs including cotton wool spots, retinal hemorrhages and Purtscher flecken. While classical Purtscher must be antedated by a traumatic incident, Purtscher-like retinopathy is used to refer to the same clinical syndrome in the absence of trauma. Various non-traumatic conditions have been associated with Purtscher-like retinopathy e.g. acute pancreatitis, preeclampsia, parturition, renal failure and multiple connective tissue disorders. In this case study, we report the occurrence of Purtscher-like retinopathy following coronary artery bypass grafting in a female patient with primary antiphospholipid syndrome (APS).

**Case presentation:**

A 48-year-old Caucasian female patient presented with a complaint of acute painless diminution of vision in the left eye (OS) that occurred approximately two months earlier. Clinical history revealed that the patient underwent coronary artery bypass grafting (CABG) two months earlier and that visual symptoms started 4 days thereafter. Furthermore, the patient reported undergoing percutaneous coronary intervention (PCI) one year before for another myocardial ischemic event. Ophthalmological examination revealed multiple yellowish-white superficial retinal lesions i.e. cotton-wool spots, exclusively in the posterior pole and predominantly macular within the temporal vascular arcades only OS. Fundus examination of the right eye (OD) was normal and the anterior segment examination of both eyes (OU) was unremarkable. A diagnosis of Purtscher-like retinopathy was made based on clinical signs, suggestive history and consolidated by fundus fluorescein angiography (FFA), spectral domain optical coherence tomography (SD-OCT) and optical coherence tomography angiography (OCTA) of macula, optic nerve head (ONH) according to the diagnostic guidelines of Miguel. The patient was referred to a rheumatologist to identify the underlying systemic cause and was diagnosed with primary antiphospholipid syndrome (APS).

**Conclusions:**

We report a case of Purtscher-like retinopathy complicating primary antiphospholipid syndrome (APS) following coronary artery bypass grafting. This conveys a message to clinicians that patients presenting with Purtscher-like retinopathy should undergo meticulous systemic work-up in order to identify potentially life-threatening underlying systemic diseases.

## Background

Purtscher retinopathy is a retinal occlusive microangiopathy that was first described by Otmar Purtscher in 1910. Purtscher described a constellation of fundoscopic abnormalities including multiple patches of retinal whitening i.e. Purtscher flecken and retinal hemorrhages. In the original case of Purtscher, presentation was predated by a fall-from-height incident [1]. Therefore, the term Purtscher retinopathy classically refers to the characteristic fundoscopic findings that occur in association with a traumatic event. Since then, multiple cases of Purtscher retinopathy have been reported as a consequence of non-ocular trauma particularly compression traumas e.g. crush injuries, chest traumas, long bone fractures [2, 3]. However, characteristic retinal signs of Purtscher retinopathy have also been frequently reported in absence of concurrent trauma. This gave rise to the term Purtscher-like retinopathy which is used to describe typical Purtscher retinopathy in absence of trauma [1]. The condition is typically bilateral, but unilateral involvement is not uncommon. Presentation is often with sudden diminution of vision a few days following trauma or the probable clinical cause for Purtscher and Purtscher-like retinopathy respectively. The most frequent fundoscopic findings are cotton-wool spots, retinal hemorrhages and Purtscher flecken of which the latter two often clear rapidly within a few weeks. Occasionally, optic disc swelling was also reported [2]. Purtscher flecken are areas of inner retinal infarcts manifesting as patches of retinal whitening mostly confined to peripapillary area and macula since these areas are the most liable to vascular occlusion due to relatively less arteriolar branching and anastomoses [2]. Cotton-wool spots represent focal accumulation of retinal nerve fiber (RNF) axonal debris secondary to infarcts of retinal nerve fiber layer (RNFL) [1]. A characteristic feature of Purtscher retinopathy is a predilection to the posterior pole particularly macular and peripapillary areas. This constituted the rationale of assigning three distinct fundoscopic zones as part of the clinical description of Purtscher retinopathy i.e. zone A centered around the fovea with a radius of 4 disc diameters, zone B extending from zone A to the equator and zone C from the equator to the ora serrata [4]. Two thirds of Purtscher retinopathy cases show fundoscopic changes exclusively in zone A .The pathogenesis underlying Purtscher retinopathy has been a contentious subject since Otmar Purtscher reported the first case in 1910, where he proposed that lympathic extravasation from retinal vasculature secondary to elevated intracranial pressure caused by head trauma in his case, was the mechanism underlying the reported fundoscopic findings [1]. The most accepted proposed theory suggests that microembolization at the level of precapillary arterioles is the core pathogenic mechanism of Purtscher retinopathy [4]. This hypothesis is supported by multiple clinical peculiarities of the condition. For example, Purtscher flecken have a characteristic polygonal configuration which corresponds to the territorial supply of a retinal capillary plexus arising from a precapillary arteriolar branch [2]. Furthermore, they show areas of demarcation that separate them from adjacent retinal arterioles which correspond to the capillary free zone [4]. This further confirms that occlusion occurs at the level of precapillary arteriolar branches. While vaso-occlusive insults in Purtscher retinopathy affect predominantly superficial inner retinal vasculature, deeper retinal capillary involvement was reported which manifested as paracentral acute middle maculopathy (PAMM) demonstrable by hyperreflectivity at the level of outer plexiform layer (OPL) and inner nuclear layer (INL) in SD-OCT and deep vascular plexus (DVP) dropouts in OCTA [5, 6]. The natural history of the disease indicates that, in 40% of cases, retinal appearance normalizes after 2 months of the onset leaving behind, nonetheless, variable degrees of inner retinal thinning, outer retinal and retinal pigment epithelial (RPE) changes [2, 5]. Various conditions have been linked to Purtscher-like retinopathy e.g. acute pancreatitis, pancreatic adenocarcinoma, preeclampsia, chronic renal failure, hemolytic uremic syndrome (HUS). Also, some cases have been associated with lymphoproliferative disorders e.g. Hodgkin lymphoma and bone marrow transplantation [7, 8]. Moreover, some viral infections e.g. SARS-COV-2 and flu-like prodromes have been reported in concurrence with Purtscher-like retinopathy [9–11]. In addition, Purtscher-like retinopathy has been reported as a complication of certain drugs e.g. tacrolimus [12]. Furthermore, the condition has been identified as a manifestation in multiple connective tissue disorders like systemic lupus erythematosus (SLE), dermatomyositis and scleroderma [13–19]. Antiphospholipid syndrome (APS) is an autoimmune disease characterized by arterial, venous or microvascular thrombosis that occurs in individuals positive for antibodies against phospholipid-binding plasma proteins i.e. antiphospholipid antibodies (aPL). The most common aPLs include lupus anticoagulants (LA), anticardiolipin antibodies (aCL) and β2 glycoprotein antibodies (β2GP) [20]. Clinical manifestations of APS include deep venous thrombosis and pulmonary embolism which are the most common. Other less common clinical presentations are arterial and venous thrombosis of the ocular vasculature e.g. central retinal vein occlusion (CRVO). Moreover, strokes and transient ischemic attacks (TIA) are the initial presentation in one quarter of APS patients. Arterial thrombosis of coronary, mesenteric and limb circulation has also been reported in APS resulting in myocardial infarctions (MI), limb or intestinal gangrene [21]. One case of Purtscher-like retinopathy was reported in association with APS in the context of preeclampsia [22]. In our case study, we report the occurrence of Purtscher-like retinopathy in association with APS in a non-obstetric condition following coronary artery bypass grafting (CABG).

## Case presentation

**Fig. 1:**
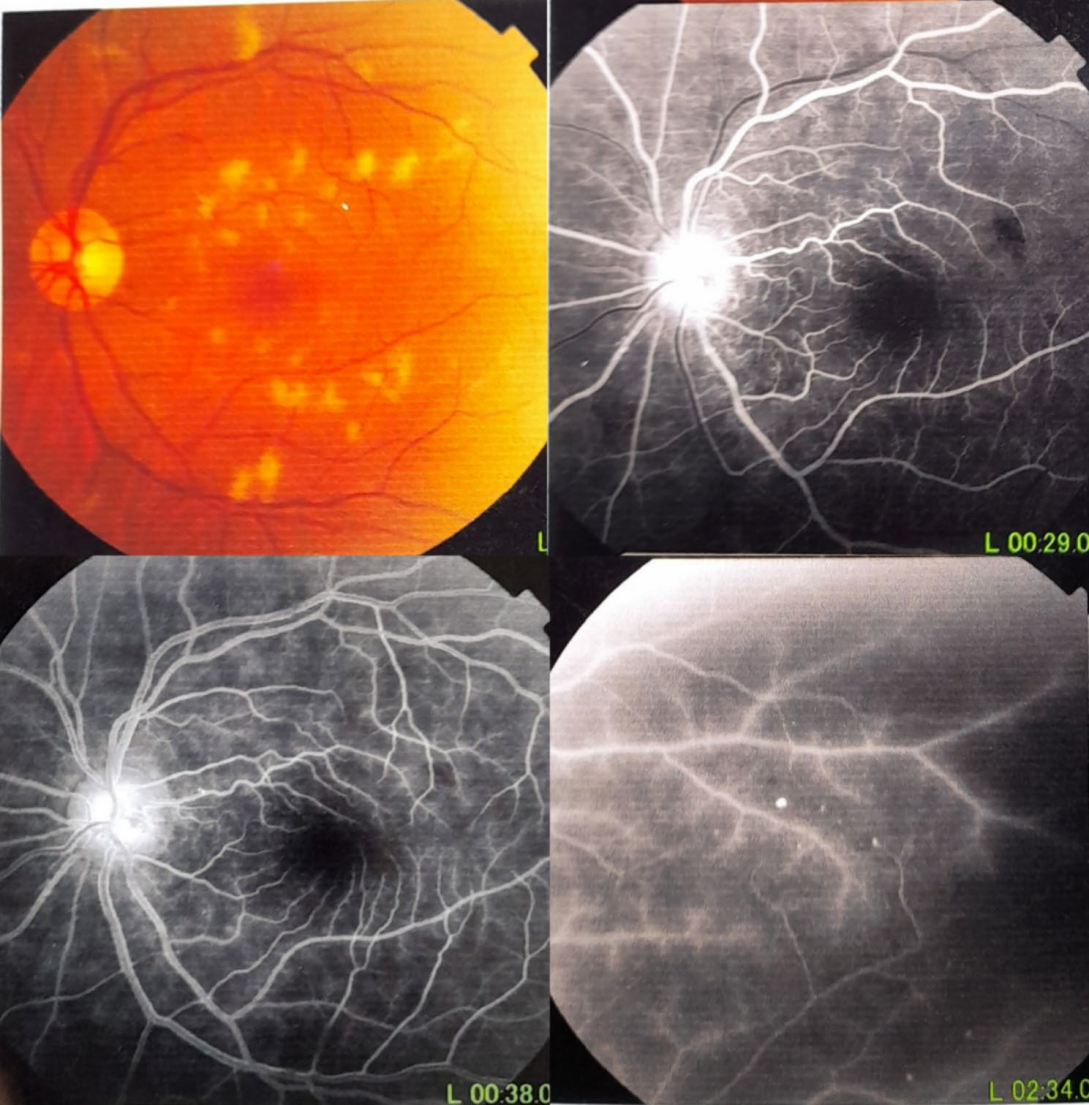
Fundus photography and fluorescein angiography OS showing posterior pole cotton-wool spots, delayed arterial and venous filling, prolonged arterio-venous (A-V) transit time, delayed choroidal filling, choroidal dropouts, peripheral retinal ischemia, widened irregular foveal avascular zone (FAZ) and late perivascular staining

**Fig. 2:**
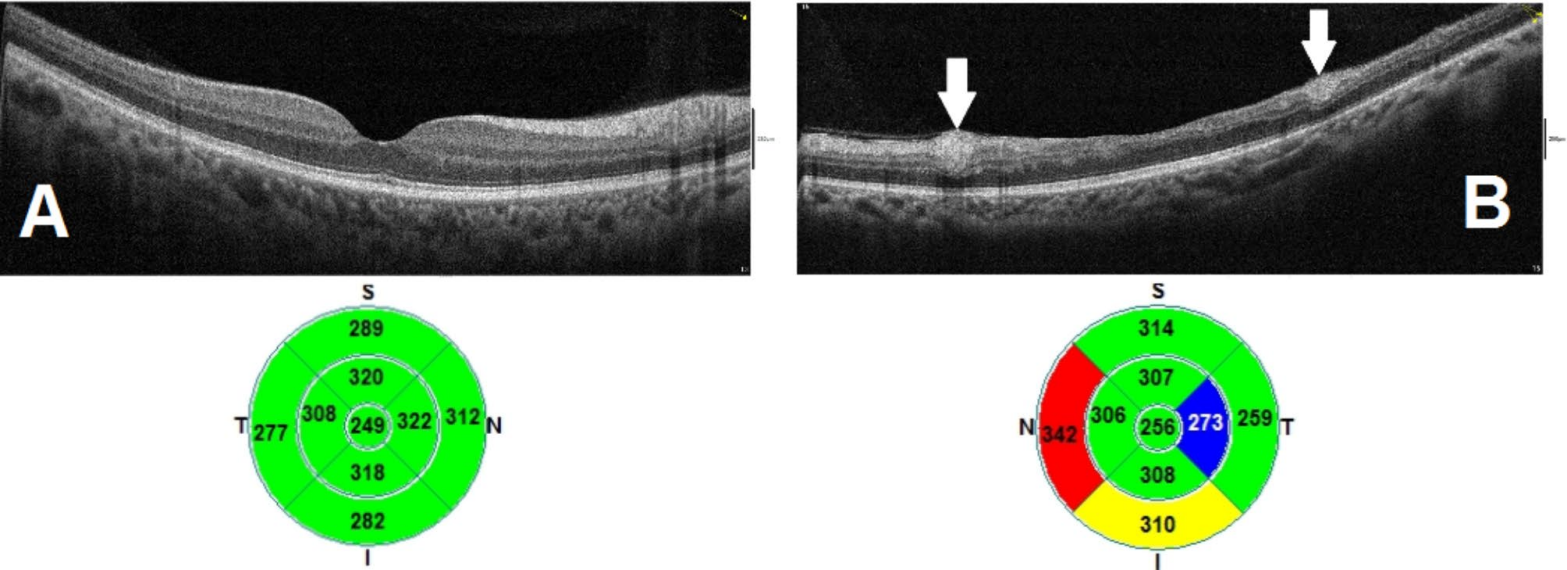
(**A**) Normal B-scan optical coherence tomography (OCT) macula and macular thickness map OD (**B**) B-scan OCT macula and macular thickness map OS showing multiple inner retinal hyperreflective lesions (white arrows) corresponding to cotton-wool spots, temporal macular thinning and nasal macular thickening.

**Fig. 3:**
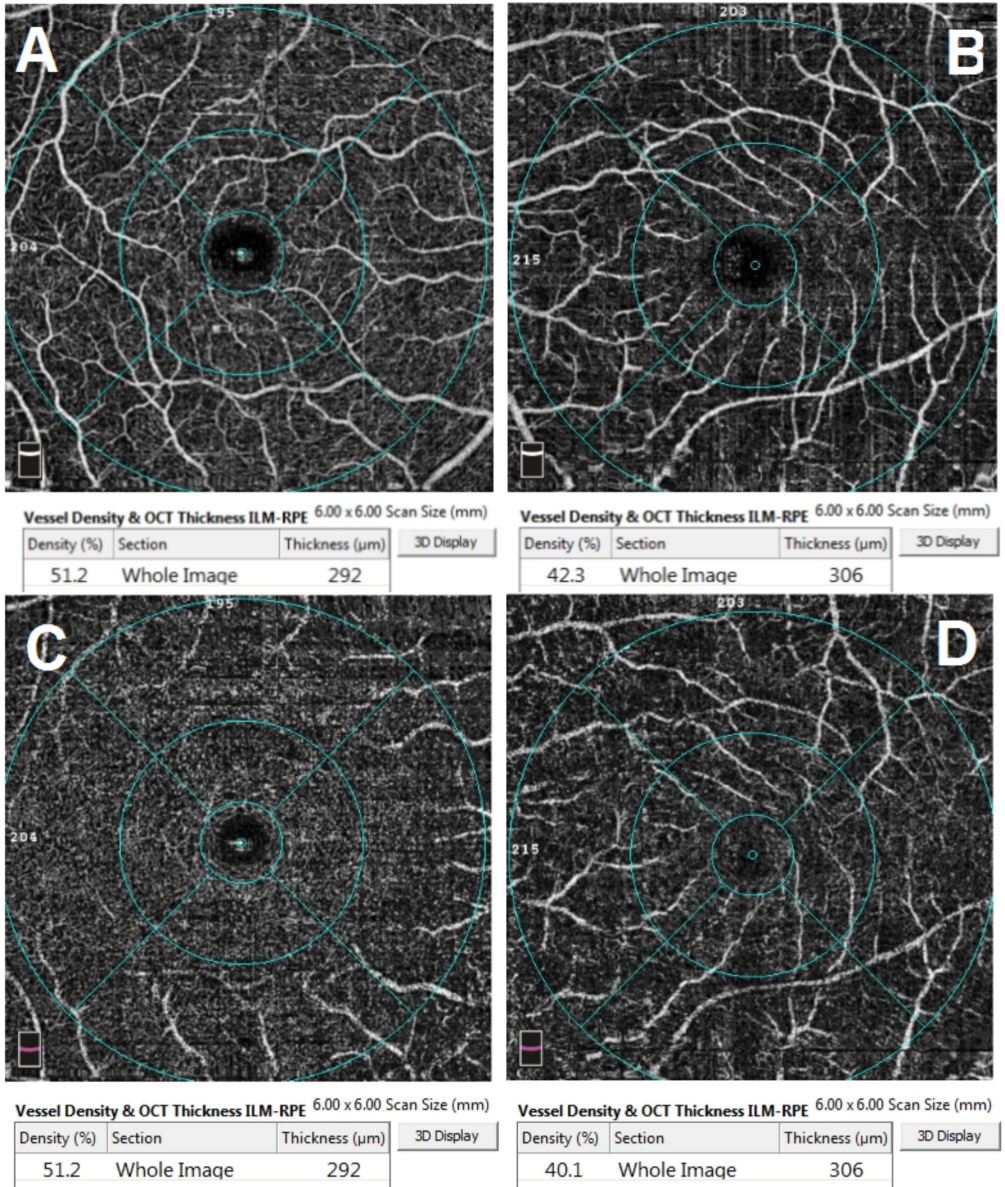
**A** and **B** Optical coherence tomography angiography (OCTA) of superficial macular vascular plexus showing reduced vascular density and capillary dropouts OS (**B**) (left ischemic maculopathy) compared to OD (**A**). **C** and **D** OCTA of the deep macular vascular plexus showing reduced vascular density and capillary dropouts OS (**D**) (left ischemic maculopathy) compared to OD (**C**).

**Fig. 4:**
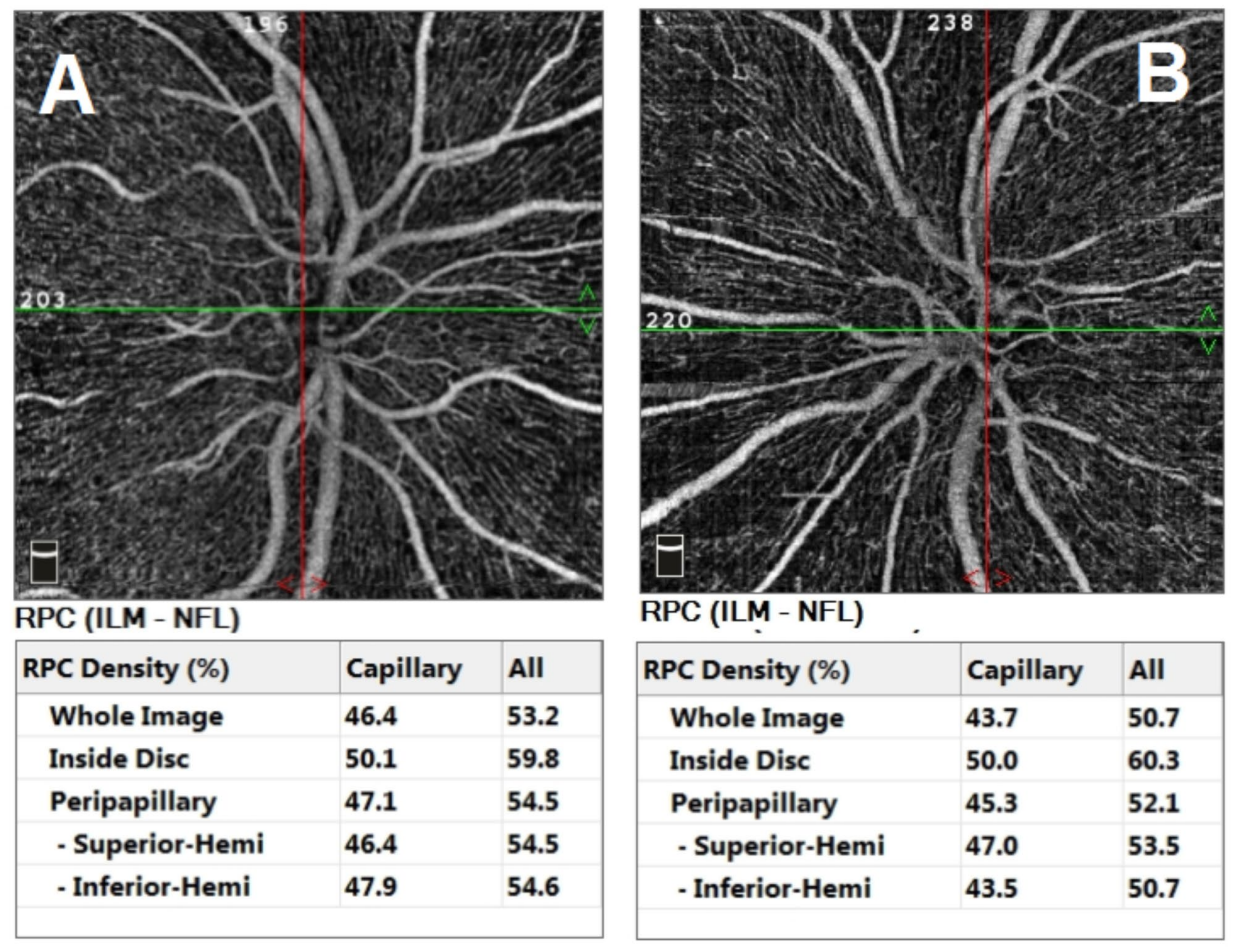
Optical coherence tomography angiography (OCTA) of optic nerve head and peripapillary area OD (**A**) and OS (**B**) showing reduced global and inferior-hemi radial peripapillary capillary density (RPCD) OS compared to OD.

**Fig. 5:**
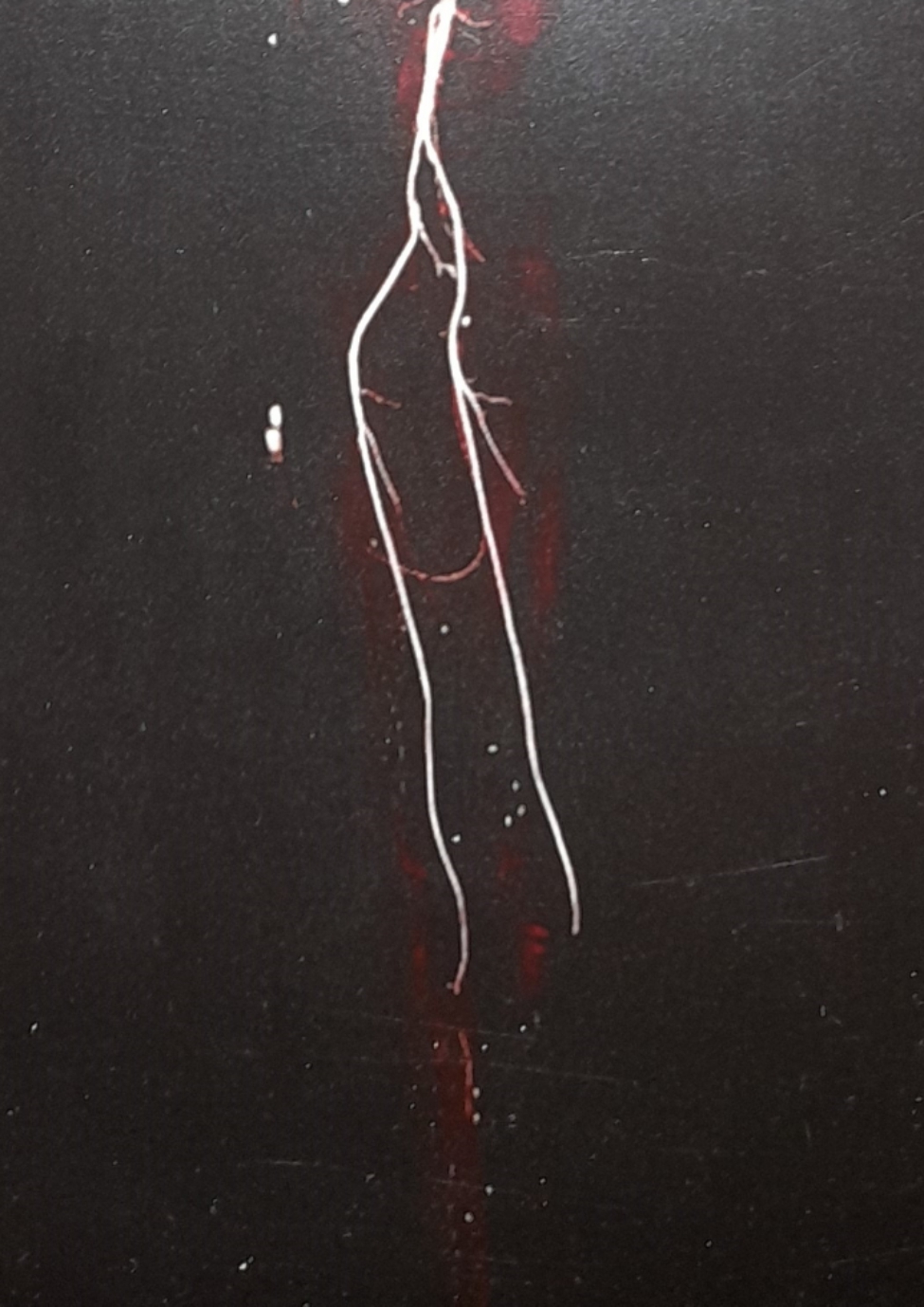
CT angiography of lower limbs showing obliteration of infrapopliteal arteries (the anterior tibial, posterior tibial and peroneal)

A 48-year-old female patient presented in December 2022 with a complaint of acute painless diminution of vision OS that occurred approximately 2 months earlier. History taking revealed that visual symptoms started four days after coronary artery bypass grafting (CABG) which was performed two months earlier for an acute myocardial ischemic (MI) event. Furthermore, the patient reported undergoing percutaneous coronary intervention (PCI) one year before for another myocardial ischemic event. The rather late presentation after nearly two months from the onset of visual symptoms was explained by the patient being admitted in the surgical intensive care unit (ICU) and inpatient ward for two weeks following CABG. At the time of presentation, BCVA was 6/6 OD and 3/60 OS. Anterior segment examination was unremarkable OU. Conversely, fundus examination OS revealed multiple yellowish-white lesions i.e. cotton-wool spots exclusively in the posterior pole. Fundoscopic examination OD was completely normal. FFA was delayed until 6 weeks after the onset of visual symptoms because the patient was admitted in the surgical ICU and inpatient ward for two weeks after CABG. Also, the cardiothoracic surgeon recommended that FFA be deferred for a few more weeks until the patient’s general condition stabilized which further contributed to the late presentation. FFA OS showed delayed arterial and venous filling, prolonged arterio-venous transit (A-V transit), patchy choroidal filling with choroidal dropouts, mid-peripheral retinal microaneurysms, peripheral retinal ischemia, widened irregular foveal avascular zone (FAZ) and late perivascular staining (Fig. 1). SD-OCT and OCTA OS were performed at presentation which revealed multiple inner retinal hyperreflectivities that corresponded to the cotton-wool spots (Fig. 2). It also showed diffuse macular thinning predominantly affecting inner retinal layers which, we hypothesize, was the sequelae of Purtscher flecken that had already resolved given the late presentation of the patient 2 months after the onset of symptoms (Fig. 2). Furthermore, OCTA showed decreased macular vascular density, optic nerve head (ONH) and radial peripapillary capillary density (RPCD) OS compared to OD (Figs. 3 and 4). Our initial differential diagnosis included central retinal artery occlusion (CRAO), branch retinal artery occlusion (BRAO), cotton-wool spots caused by surgery-related anemia or bacteremia, retinitis and Purtscher-like retinopathy. However, CRAO and BRAO were discarded on basis of patchy, multifocal, discrete yellowish-white inner retinal lesions as opposed to the diffuse or sectoral retinal whitening characteristic of CRAO and BRAO respectively. Anemia and bacteremia were excluded because complete blood count (CBC) showed only mild microcytic hypochromic anemia with blood hemoglobin level of 10.2 gm/dl not sufficient to cause extensive cotton-wool spots. Also, CBC was negative for leukocytosis and bandemia and there was no history of fever which are important markers of bacteremia. In addition, retinitis was excluded due to the relatively poor vision that was inconsistent with the foveal-sparing lesions, were they to be retinitis and also on the basis of absence of inflammatory signs in both the posterior and anterior segments. Moreover, the angiographic findings of delayed arterial and venous filling and prolonged arteriovenous (A-V) transit time highly suggested a vascular rather than an inflammatory etiology particularly in a patient with history of two episodes of acute myocardial ischemia. Therefore, a diagnosis of Purtscher-like retinopathy was made based on the multiple cotton-wool spots with the exclusive posterior pole distribution, the supporting FFA and SD-OCT findings elaborated earlier as well as the presence of a suggestive concurrent systemic cause i.e. APS which was diagnosed later. This is consistent with the diagnostic criteria of Purtscher retinopathy proposed by Miguel et al. where a diagnosis of Purtscher retinopathy is made when a patient has at least three of the five stated criteria which include Purtscher flecken, retinal hemorrhages in low to moderate number, cotton-wool spots confined to the posterior pole, a probable explanatory association and complementary investigations compatible with the diagnosis [2]. Our case fulfills three of these criteria including the cotton-wool spots confined to the posterior pole, the probable cause which is APS and the compatible investigative findings in FFA, SD-OCT and OCTA [5, 6]. We also hypothesize that the patient probably had Purtscher flecken, that had nonetheless resolved at the rather late time of presentation 2 months after the onset of symptoms. However, the marked inner retinal thinning highly suggested that Purtscher flecken may have indeed developed at an earlier stage of the disease. A diagnosis of Purtscher-like retinopathy in a young female patient with systemic manifestations of occlusive vasculopathy necessitated further evaluation for an underlying systemic cause that, we suggest, was the causative factor of both the myocardial ischemia and the Purtscher-like retinopathy. So, the patient was referred to a rheumatologist who diagnosed her with primary APS based on the arterial thrombotic events i.e. myocardial infarctions, retinal microvascular thrombosis i.e. Purtscher-like retinopathy, thrombosed segments of radial, ulnar, anterior tibial, posterior tibial and peroneal arteries in arterial duplex and CT angiography (Fig. 5) and positive anticardiolipin antibodies (aCL) according to the updated Sapporo classification of APS [23]. Other laboratory investigations, performed by the rheumatologist, included CBC, liver enzymes i.e. alanine transaminase (ALT) and aspartate transaminase (AST), kidney function tests i.e. serum creatinine and blood urea, rheumatoid factor (RF), antinuclear antibodies (ANA), anti-double-stranded DNA antibodies (dsDNA Abs), β2GP and LA. RF, ANA and dsDNA Abs were negative excluding SLE which is a common cause of secondary APS. CBC was positive for mild microcytic hypochromic anemia with other parameters being within normal ranges. Also, liver enzymes and kidney function tests were normal. Despite β2GP and LA being negative, persistently high aCL together with the aforementioned clinical signs supported a diagnosis of primary APS according to the updated Sapporo classification of APS [23]. Consequently, the patient was hospitalized and received anticoagulants, pulsed steroid therapy and cyclophosphamide. In conclusion, the patient was diagnosed as Purtscher-like retinopathy secondary to APS which may have been precipitated by CABG.

## Discussion and conclusions

**Fig. 6:**
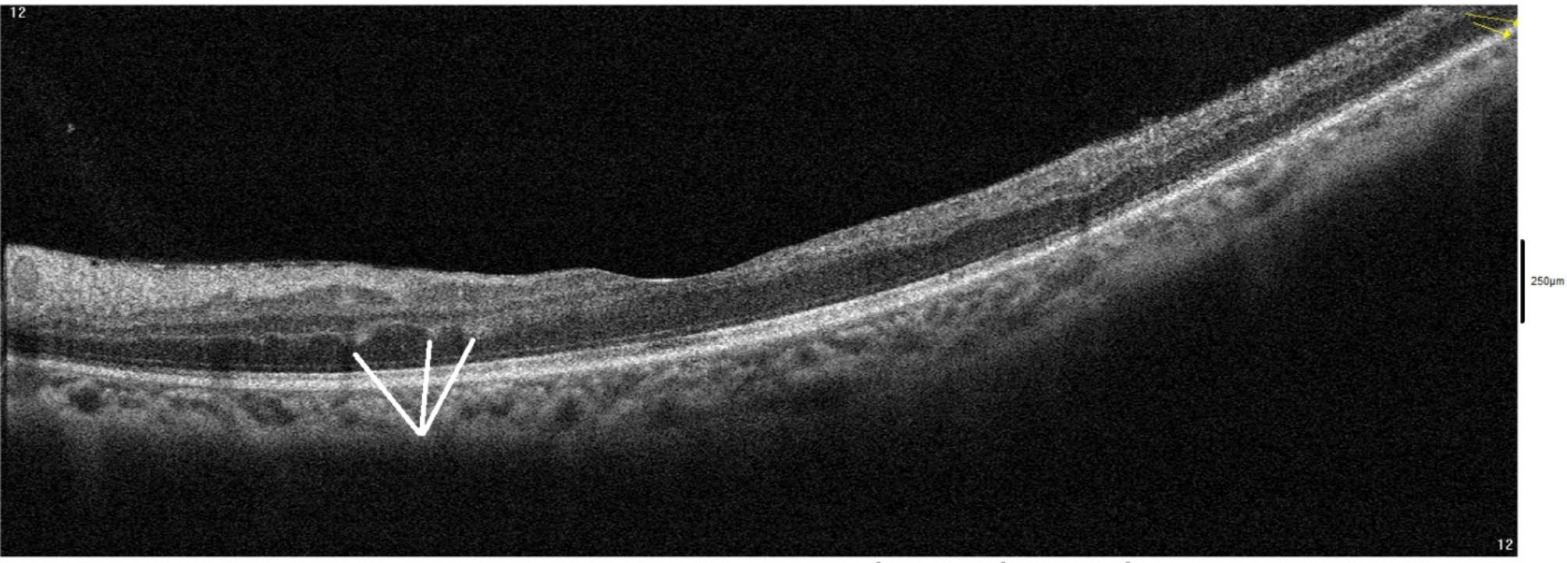
B-scan optical coherence tomography macula OS showing irregularity and hyperreflectivity at the level of outer plexiform layer (OPL) (white lines) signifying paracentral acute middle maculopathy (PAMM).

Purtscher and Purtscher-like retinopathy are rare retinal vascular occlusive disorders that affect precapillary arteriolar branches and cause painless diminution of vision [4]. They manifest with a characteristic fundoscopic appearance comprising Purtscher flecken, cotton-wool spots and retinal hemorrhages. Pathophysiologically, vascular occlusion has been attributed to different mechanisms according to the underlying cause. In traumatic cases, it is generally suggested that fat microemboli, released from traumatized tissue into the systemic circulation, and vasculitis induced by free fatty acids and lipases are responsible for vascular occlusion [3, 24, 25]. On the other hand, in cases related to acute pancreatitis and pancreatic adenocarcinoma, it is proposed that disseminated pancreatic proteases are the culprit [2, 13, 14]. Alternatively, the formation of leukocytic aggregates, precipitated by activation of the complement system, is hypothesized to be the pathogenic mechanism in cases linked to connective tissue disorders and lymphoproliferative conditions [26, 27]. For the clinical diagnosis of Purtscher retinopathy, Miguel et al. formulated diagnostic guidelines that comprised five criteria i.e. Purtscher flecken, posterior pole cotton-wool spots, low to moderate number retinal hemorrhages, a probable associated cause and supporting investigative findings [2]. The investigations of importance in Purtscher retinopathy include FFA and SD-OCT. In our case, FFA OS showed delayed arterial filling, prolonged A-V transit time, widened FAZ, peripheral retinal ischemia with late perivascular staining which corresponded to the ischemic vaso-occlusive nature of the condition. In the SD-OCT-macula of our patient, there was thinning of the inner retina particularly temporally which, we hypothesize, was the sequelae of resolved Purtscher flecken (Fig. 2). Conversely, nasal macular thickening was observed which was due to the RNFL infarct i.e. cotton-wool spots given the fact that RNFL thickness is highest in the peripapillary area (Fig. 2). The heterogeneity of the macular thickness map with temporal thinning and nasal thickening was one of the reasons to exclude CRAO which is known to cause diffuse retinal thinning. Additionally, OPL and INL hyperreflectivity and irregularity were evident in the SD-OCT-macula signifying deep vascular plexus (DVP) involvement which is termed PAMM (Fig. 6). DVP involvement was further confirmed by reduced deep vascular density (DVD) in OCTA (Fig. 3) [5]. Furthermore, OCTA-macula demonstrated reduced superficial vascular density (SVD) and capillary dropouts that resulted from microvascular occlusion and capillary nonperfusion (Fig. 3). Similarly, OCTA of the optic nerve head and peripapillary area demonstrated reduced vascular density OS compared to OD (Fig. 4). The importance of identifying Purtscher-like retinopathy as a distinct clinical entity lies in the potentially serious and life-threatening associated conditions. While multiple connective tissue diseases have been associated with Purtscher-like retinopathy e.g. SLE, scleroderma and dermatomyositis [17, 18], this is the first case report of an association between primary APS and Purtscher-like retinopathy in a non-obstetric context i.e. CABG. We hypothesize that cardiopulmonary bypass which is used during CABG was the precipitating factor for the retinal microvascular thrombosis manifesting as Purtscher-like retinopathy. Procoagulant changes have been reported with cardiopulmonary bypass as well as major cerebral and cardiothoracic surgeries and are expected to be more frequent in patients with hypercoagulable states like APS [28–30]. Only one case of Purtscher-like retinopathy was previously reported in association with APS but in an obstetric patient with pre-eclampsia [22]. This stresses the importance of differentiating Purtscher-like retinopathy from other conditions with similar presentations like hypertensive retinopathy since the former necessitates extensive systemic work-up to identify a potentially serious underlying systemic cause. It also warrants the inclusion of primary APS in the lists of probable causes of Purtscher-like retinopathy independent of obstetric conditions e.g. preeclampsia and eclampsia which further widens our clinical perspective of the systemic causes of such a rare retinal presentation and improves the standard of care provided to these patients.

## Data Availability

Data is available from the corresponding author on reasonable request.

## List of Abbreviations

ALT: Alanine Transaminase
ANA: Antinuclear Antibody
AST: Aspartate Transaminase
APS: Antiphospholipid Syndrome
aCL: Anticardiolipin Antibody
A-V: Arterio-Venous
β2GP: β2 Glycoprotein Antibody
BCVA: Best Corrected Visual Acuity
BRAO: Branch Retinal Artery Occlusion
CABG: Coronary Artery Bypass Grafting
CBC: Complete Blood Count
CRAO: Central Retinal Artery Occlusion
DVP: Deep Vascular Plexus
DVD: Deep Vascular Density
FAZ: Foveal Avascular Zone
FFA: Fundus Fluorescein Angiography
HUS: Hemolytic Uremic Syndrome
INL: Inner Nuclear Layer
LA: Lupus Anticoagulant
MI: Myocardial Ischemia
OCTA: Optical Coherence Tomography Angiography
OD: Oculus Dexter (Right Eye)
ONH: Optic Nerve Head
OPL: Outer Plexiform Layer
OS: Oculus Sinister (Left Eye)
OU: Oculus Uterque (Both Eyes)
PAMM: Paracentral Acute Middle Maculopathy
PCI: Percutaneous Coronary Intervention
RF: Rheumatoid Factor
RNF: Retinal Nerve Fiber
RNFL: Retinal Nerve Fiber Layer
RPCD: Radial Peripapillary Capillary Density
RPE: Retinal Pigment Epithelium
SD-OCT: Spectral Domain Optical Coherence Tomography
SLE: Systemic Lupus Erythematosus
SVD: Superficial Vascular Density
SVP: Superficial Vascular Plexus
